# Inhibition of EED activity enhances cell survival of female germline stem cell and improves the oocytes production during oogenesis *in vitro*

**DOI:** 10.1098/rsob.220211

**Published:** 2023-01-25

**Authors:** Jiapeng Wang, Junxian Fang, Mingqian Feng, Liping Li, Lixin Ma, Xiaorong Zhao, Yanfeng Dai

**Affiliations:** College of Life Sciences, Inner Mongolia University, Xilingol South Road No. 49, Hohhot 010020, People's Republic of China

**Keywords:** female germline stem cell, ovarian organoids, EED, H3K27me3, OCT4, oogenesis *in vitro*

## Abstract

Ovarian organoids, based on female germline stem cells (FGSCs), are nowadays widely applied for reproductive medicine screening and exploring the potential mechanisms during mammalian oogenesis. However, there are still key issues that urgently need to be resolved in ovarian organoid technology, one of which is to establish a culture system that effectively expands FGSCs *in vitro*, as well as maintaining the unipotentcy of FGSCs to differentiate into oocytes. Here, FGSCs were EED226 treated and processed for examination of proliferation and differentiation *in vitro*. According to the results, EED226 specifically increased FGSC survival by decreasing the enrichment of H3K27me3 on *Oct4* promoter and exon, as well as enhancing OCT4 expression and inhibiting P53 and P63 expression. Notably, we also found that FGSCs with EED226 treatment differentiated into more oocytes during oogenesis *in vitro*, and the resultant oocytes maintained a low level of P63 versus control at early stage development. These results demonstrated that inhibition of EED activity appeared to promote the survival of FGSCs and markedly inhibited their apoptosis during *in vitro* differentiation. As a result of our study, we propose an effective culture strategy to culture FGSCs and obtain oocytes *in vitro*, which provides a new vision for oogenesis *in vitro*.

## Graphical abstract

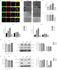

## 1. Introduction

EED, SUZ12, EZH1/2 and RbBP4/7 are the four main subunits of Polycomb-repressive complex 2 (PRC2), which regulates the enrichment of di- and trimethylation of histone H3 Lys27 (H3K27me2/3) [1]. Despite the fact that EZH2 is a central catalytic domain of PRC2 [2], EED is required for physically binding H3K27me3 via five tandemly repeated WD motifs, and hence exerts an important function in PRC2 assembly [3,4]. Inactivation of either EED [5] or EZH2 [6] severely compromises the core function of PRC2 and further causes loss of H3K27me3. Indeed, PRC2 regulates the stability of gene expression *in vivo* by promoting or blocking cell differentiation, fine-tuning cell fate decisions and guiding cell differentiation throughout the shift from pluripotent to differentiation [7].

In the fetal ovary, the gain of H3K27me3 was initially identified in primordial germ cells (PGCs) at E10.5 and persisted at its peak level until E15.5. EED and EZH2 were all detected in the nuclei of E11.5 and E12.5 PGCs, then they continued to be detectable until E15.5 [8]. In addition, H3K27me3, EED and EZH2 also were likewise abundant in growing oocytes of postnatal day mouse ovaries [9]. H3K27me3 was shown to be elevated in stage-specific genes relevant to meiotic development during mammalian spermatogenesis [10]. The conditional deletion of EED in the male germ cell results in complete male infertility [10]. Female EED deletion mice, on the other hand, had normal fertility and generated pups with considerable overgrowth [11]. By contrast, the conditional knockout of EZH2 in growing oocytes remained normal reproductive characteristics, and the pups were born underweight [12].

The functional study of female germline stem cells (FGSCs) has significant implications for our comprehension of oogenesis [13]. At present, the way to enhance the FGSC proliferation efficiency is a major focus of the following research. Previous study reported that GDNF [14] significantly contributes to FGSC self-renewal, creating opportunities for gametogenesis of mammals *in vitro* [15]. Recently, Grosswendt *et al.* [16] reported that PRC2 plays a vital role in mouse early embryonic development stage, in which EED deficiency in zygotic leads to embryonic lethality in mice by impairing gastrulation development. However, EED mutants have substantially more PGCs, indicating that PRC2 dominates the limitation in early germline lineage. This further implicated that inhibition of PRC2 may promote FGSC proliferation and survival.

In the present experiment, we systematically examined the function of PRC2 in FGSC proliferation and differentiation *in vitro*. Our results indicated that FGSCs with EED226 treatment maintained a significantly higher survival during *in vitro* cultivation, which has shed new light on FGSC culturing strategy.

## 2. Results

### 2.1. Inhibition of the PRC2 function could boost FGSC proliferation

**Figure 1.**
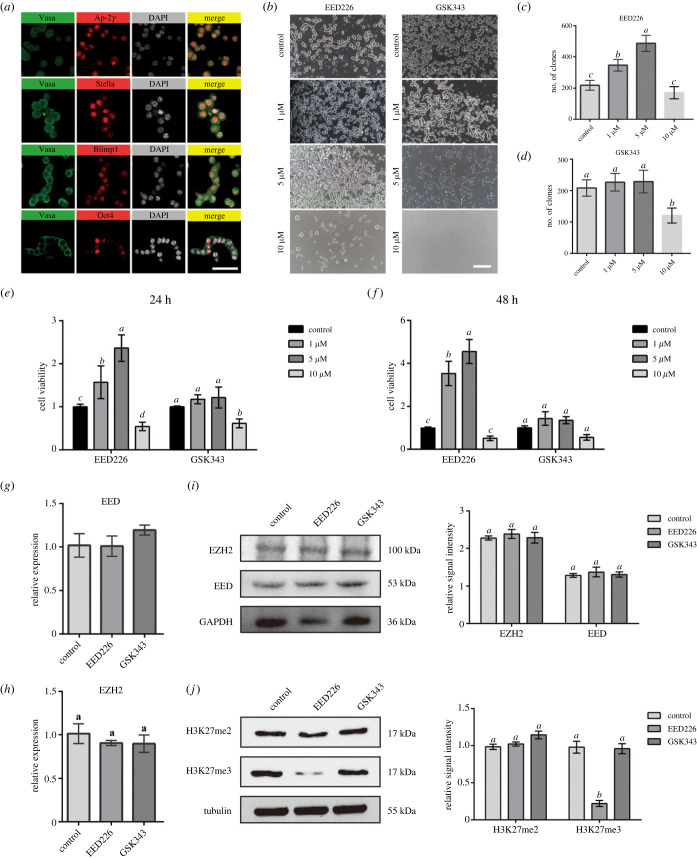
EED226 enhanced the FGSC proliferation and decreased the accumulation of H3K27me3. (*a*) Immunofluorescence for AP-2*γ*, STELLA, BLIMP1, OCT4 and VASA in FGSC, DNA (DAPI) is shown in grey. Scale bar, 5 μm. (*b*) FGSC colony formation assay in different concentrations of EED226 and GSK343. Scale bar, 100 µm. (*c*) Quantification analyses of colony formation assay in EED226 group. Data are shown as mean ± s.d. Significant difference according to one-way ANOVA. (*d*) Quantification analyses of colony formation assay in GSK343 group. Data are shown as mean ± s.d. Significant difference according to one-way ANOVA. (*e,f*) Quantification analyses of CCK-8 assays to detect cell viability of FGSCs treated with different concentrations of EED226 and GSK343 for 24 h and 48 h. Data are shown as mean ± s.d. Significant difference according to one-way ANOVA. (*g,h*) The expressions of EED and EZH2 genes were quantified using a standard quantitative PCR in three groups. Data are shown as mean ± s.d. Significant difference according to one-way ANOVA. (*i*) EZH2 and EED protein expression detected by western blot and quantification of western blot. GAPDH was used as loading control. Quantification of western blot results (right). Data are shown as mean ± s.d. Significant difference according to one-way ANOVA. (*j*) H3K27me2/3 protein expression detected by western blot and quantification of western blot. Tubulin was used as loading control. Quantification of western blot results (right). Data are shown as mean ± s.d. Significant difference according to one-way ANOVA.

Firstly, FGSCs were identified by germline (AP-2*γ*, BLIMP1, STELLA and VASA) and pluripotency (OCT4) specific gene expression. Immunofluorescence analysis showed positivity for nuclear AP-2*γ*, BLIMP1, STELLA, OCT4 and cytoplasmic VASA proteins in isolated FGSCs (figure 1*a*). Then, to probe the role of PRC2 in FGSC self-renewal, we evaluated and examined the effect of small chemicals (EED226 and GSK343) treatment on FGSC growth via colony formation array (figure 1*b*). The result demonstrated that EED226 significantly enhanced the formation of colonies and the growth of cells in a dose-dependent manner (1, 5 µM) (figure 1*c*). However, it is here observed that when the EED226 concentrations added were higher (10 µM), the clones number was markedly decreased compared with the clonal efficiency of other groups. What calls for special attention is that there appeared to be no significant difference in the number of the FGSC clones with GSK343 treatment (figure 1*d*). To further confirm this effect, we conducted a time gradient assay to evaluate the FGSC proliferative effect of inhibitors at various concentrations by CCK8 detection. The result indicated that the OD values of FGSCs with 5 µM EED226 treatment markedly increased relative to controls at 24 h and 48 h (figure 1*e*). Likewise, CCK8 assay results showed that GSK343 had no significant effect on FGSC proliferation (figure 1*f*). EdU incorporation assay was further performed to validate the facilitation effects of EED226 on FGSC proliferation. Significantly, more EdU incorporation was induced by the EED226 treatment, compared with untreated controls (electronic supplementary material, figure S1). Taken together, the above results demonstrate that 5 µM EED226 was able to enhance FGSC proliferation.

Considering that EED226 and GSK343 are specific inhibitors of EED and EZH2, respectively, we next examined the expression of EED and EZH2 in FGSCs after treatment with EED226 or GSK343 via RT-qPCR and WB. Results indicated that no significant differences in EED and EZH2 expression were observed after the addition of inhibitors (figure 1*g–i*). Correspondingly, to examine the effect of inhibitors on the enrichment of H3K27me2/3, we compared the level of H3K27me2/3 in FGSCs after with or without inhibitor treatment. There were no obvious changes in the level of H3K27me2 regardless of the addition of EED226 or GSK343 (figure 1*j*). As well, H3K27me3 levels did not significantly change when FGSCs were exposed to GSK343, but the level of H3K27me3 was markedly reduced after EED226 treatment (figure 1*j*). Based on above results, EED226 potently binds EED *in vitro*, inhibited PRC2 catalytic capability in consequence decreasing H3K27me3 level, and promoted FGSC proliferation.

### 2.2. Inhibition of the EED activity promotes the expression of OCT4 and inhibits the expression of P53 and P63

**Figure 2.**
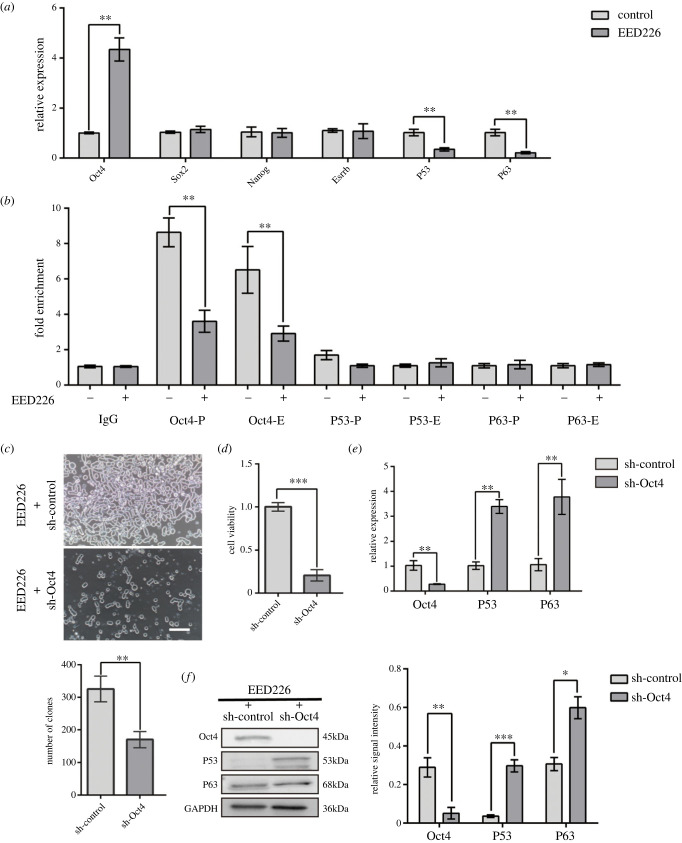
EED226 regulated the H3K27me3 modification on *Oct4* promoter and exon. (*a*) RT-qPCR analysis for *Oct4*, *Sox2*, *Nanog*, *Esrrb*, *P53* and *P63* in FGSCs treated with or without EED226. Data are shown as mean ± SEM. **p < 0.01, according to Student's *t* test. (*b*) ChIP-qPCR assay was used to measure the enrichment of H3K27me3 on the *Oct4*, *P53*, *P63* promoter/exon in FGSCs at the indicated status. IgG served as negative control. Data are shown as mean ± SEM. **p < 0.01, according to Student's *t* test. (*c*) Colony formation assays were performed to determine the proliferation of FGSCs with *Oct4* knockdown. Scale bar, 100 µm. Quantitative data are shown as mean ± s.d. **p < 0.01, according to Student's *t* test. (*d*) Graphic representation of results from CCK-8 assays to determine cell viability of FGSCs with *Oct4* knockdown. Data are shown as mean ±SEM. ***p < 0.001, according to Student's *t* test. (*e*,*f*) RT-qPCR and western blotting analyses of the OCT4, P53 and P63 in FGSCs with *Oct4* knockdown post indicated treatments. Quantitative data are shown as mean ± s.d. *p < 0.05, **p < 0.01, ***p < 0.001 according to two-tailed Student's *t* test.

To investigate the transcriptional effects of EED inhibition, we detected the expression level of survival-associated genes in FGSCs. The expression of *Oct4* was significantly enhanced with EED226 treatment. Also, *P53* and *P63* expressions, as indicators of apoptosis, were obviously downregulated in comparison to the control group (figure 2*a*, *p* < 0.01), suggesting that the EED226-mediated epigenetic dynamics of H3K27me3 might be correlated with the transcriptional variation of these genes. We further verified this interaction via ChIP-qPCR analysis. It turned out that H3K27me3 occupancy at the *Oct4* promoter and exon region decreased after EED226 treatment (figure 2*b*, *p* < 0.01). This corresponded with its expression levels. As for *P53* and *P63*, we did not detect the variation of H3K27me3 modifications at the promoter and exon regions. These findings suggested that EED226 could regulate the H3K27me3 enrichment on the *Oct4* promoter and exon. On the other hand, *Oct4* may be core mediator of the effect of EED226.

To verify the above deduction, we employed RNA interference strategy to generate the *Oct4* knockdown FGSCs. First, we examined the cellular proliferation of FGSCs with *Oct4* knockdown by the colony formation and CCK8 assay in the presence of EED226. The results indicated that the survival ability of FGSCs was significantly reduced with *Oct4* knocked down in comparison to the control group (figure 2*c*, *p* < 0.01, figure 2*d*, *p* < 0.001). Besides, RT-qPCR and western blot results confirmed that the expression level of OCT4 was decreased (figure 2*e,f*, *p* < 0.01), accompanied by a concurrent elevation of P53 and P63 expression. Those results indicated the effect of EED226 on FGSC survival depends on the expression of OCT4. But meanwhile, the results indicated that there may be a negative regulatory relationship between OCT4 and expression of P53 and P63.

### 2.3. Inhibition of EED function does not affect the differentiation capacity of FGSCs *in vitro*

**Figure 3.**
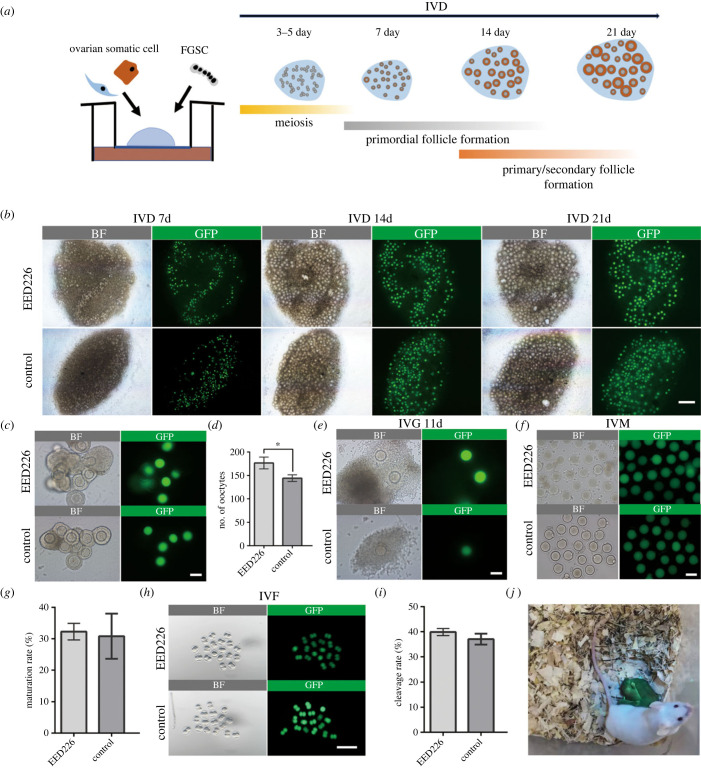
Oocyte production from FGSCs *in vitro*. (*a*) Schematic diagram of the generation of ovarian organoids. (*b*) Representative images of organoids differentiated from FGSCs treated with EED226 or vehicle control at IVD 7 d, 14 d and 21 d. BF = bright field. Scale bar, 500 *μ*m. (*c*) Statistics on the number of primary/secondary-like follicles (P/S). Data are shown as mean ± SEM. **p < 0.01, according to two-tailed Student's *t* test. (*d,e*) Representative images of single follicles during IVG culture. Scale bar, 100 µm. (*f*) Representative images of ovarian organoid-derived mature oocytes after *in vitro* maturation. Scale bar, 100 µm. (*g*) The statistics for the maturation rate in three groups. Data are shown as mean ± s.d. **p < 0.01, according to two-tailed Student's *t* test. (*h*) Representative images of ovarian organoid-derived mature oocytes after *in vitro* fertilization. Scale bar, 200 μm. (*i*) *In vitro* fertilization rate of ovarian organoid-derived mature oocytes. Data are shown as mean ± s.d. **p < 0.01, according to two-tailed Student's *t* test. (*j*) Representative offspring derived from EED226 group.

In the above experiments, the regulatory role of EED on FGSC proliferation has been established. Next, we systematically characterized the germline of FGSCs after EED inhibition via the reconstitution system. FGSCs were aggregated with ovarian somatic cells to produce rOvaries and cultured under Transwell (figure 3*a*). The close observation of IVD culture revealed that follicles from GFP^+^ FGSCs (from *β*-actin-GFP mice ovaries) were formed around 7 days of culture. After 21 days of culture, FGSCs differentiated to form primary/secondary-like follicle structures in both groups (figure 3*b*). Subsequently, we counted the number of GFP^+^ oocytes isolated from rOvaries in both groups. On average, 172.67 ± 12.67 GFP^+^ oocytes were formed per rOvary, which was significantly increased compared with control (144.33 ± 7.10, *p* < 0.05) (figure 3*c,d*). According to the above results, a significant number of oocytes were produced from the EED226 group under the identical culture conditions employed, which may be relevant to the high survival efficiency of FGSCs after being induced by EED226. Next, individual follicles were performed in IVG culture. Following 11 days of culture, primary oocytes grew to germinal vesicle oocytes in both groups (figure 3*e*). After maturation, the percentage of *in-vitro*-generated MII oocytes was 32.67 ± 2.24% in EED226 group, and no significant difference was observed versus the control group (30.82 ± 7.22%, *p* > 0.05, figure 3*f,g*). Then, *in-vitro*-generated MII oocytes were conducted to *in vitro* fertilization (IVF). By IVF, oocytes were developed to two-cell embryos (39.90 ± 1.42% versus 37.06 ± 2.15%, *p* > 0.05, figure 3*h,i*). Three (4.41%) out of 68 two-cell embryos transferred to pseudopregnant ICR females successfully produced pups in the EED226 group (figure 3*j*). Compared with control, no distinctive offspring body weight changes were observed in the EED226 group (electronic supplementary material, figure S2). Mating experiments were used to assess the fertility of progeny derived from ovarian organoids. When mated with normal males or females of proven fertility, the respective adults derived from EED226 organoid oocytes produced similar litter sizes compared with control group (electronic supplementary material, table S4). These results showed that ovarian organoids based on EED226 system were capable of generating fertile offspring.

### 2.4. Inhibition of the function of EED did not affect the process of meiosis

**Figure 4.**
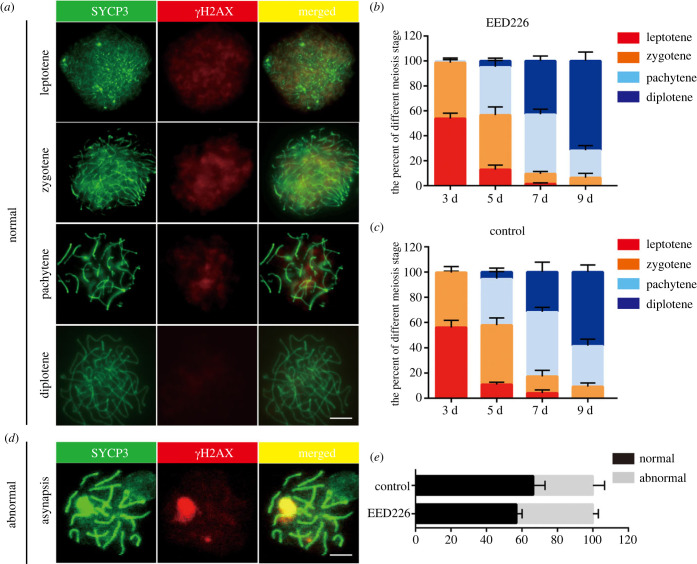
Meiotic progressions in ovarian organoids. (*a*) Representative images of each stage of meiotic prophase I in IVD culture. Scale bar, 10 μm. (*b,c*) Percentages of each stage in EED226 and control groups are shown. (*d*) Asynapsis of meiotic chromosome at the pachytene stage in IVD culture. Scale bar, 10 μm. (*e*) The percentage of asynapsis in cells at the pachytene stages in four rOvaries or three 1–3 dpp gonads cultured for 5 days.

Meiotic recombination is a highly complex process required for oogenesis. We analysed prophase I progression in rOvaries, including leptotene, zygotene, pachytene and diplotene. Oocytes from rOvaries exhibited the four meiosis markers on IVD days 3 to 9, which indicated FGSCs were induced to enter meiosis on IVD day 3 (figure 4*a*). In addition, we also compared the percent of prophase I progression in rOvaries from two FGSC sources. Furtherly, statistical analysis showed no statistical difference between EED226 and control groups, suggesting that their ability to enter meiosis were equivalent (figure 4*b,c*). As observed by the distribution of *γ*H_2_AX on the pachytene chromosomes, persistent double-strand breaks are seen in *in-vitro*-generated oocytes. Of note, by pachytene stage, nearly 43.02 ± 3.10% and 33.57 ± 6.61% of oocytes in EED226 and control groups display asynapsis to some extent, a percentage significantly greater than that ever observed in the EED226 group (10%) (figure 4*d,e*).

**Figure 5.**
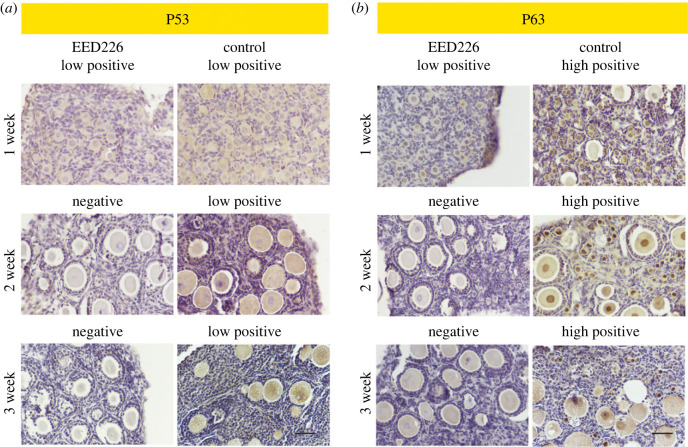
The treatment of EED226 inhibited the expression of P63 in ovarian organoids. Immunohistochemical detection of (*a*) P53 and (*b*) P63 in ovarian organoids during IVD culture. Scale bar, 100 µm.

The finding of the high degree of asynapsis in the EED226 group was understandable and acceptable, given that P63 and P53 are thought to play roles in a conserved mechanism for controlling meiosis integrity [17] and given the recent observation that P63 and P53 presented low expression in FGSCs with EED226 treatment. Then, the expressions of P53 and P63 were detected at different time periods (IVD 7, 14 and 21 d) by IHC. Moreover, P63 and P53 are specifically highly expressed in oocyte nucleus and cytoplasm, respectively. Quantitative analysis results indicate that the intensity of P53 was low positive or negative from IVD 7 d to 21 d in EED226 and control groups (figure 5*a*). Meanwhile, in the control group, the intensity of P63 expression begin to increase from IVD 7 d. After IVD 14 and 21 d, the expression levels of P63 remain high in the oocytes of primordial, primary and early secondary-like follicles. However, the intensity of P63 was lower in the EED226 group in comparison with the control (figure 5*b*).

## 3. Discussion

A previous study revealed that disruption of EED, as the core PRC2 subunit, significantly elevates the population of PGCs [16]. Based on this, we speculate that the functional inhibition of PRC2 may contribute to the proliferation of FGSCs *in vitro*. Here, the potential effects of EED226 and GSK343, as the inhibitors of EED and EZH2, on the *in vitro* proliferation and developmental competence of FGSCs were analysed in this study for the first time. Our results show that 5 µM EED226 effectively promoted cell survival via downregulating the occupancy of H3K27me3 on *Oct4* gene regions and further increased the expression of OCT4. Moreover, we found that FGSCs treated with EED226 could develop into oogonia that entered meiosis and successfully differentiate into functional oocytes *in vitro*.

When germ cell destiny was established in E7.5, transcriptional regulation during development is governed by the dynamic of H3K27me3 enrichment [18]. H3K27me3 is enriched at developmental gene promoter region in PGCs [1,19], whereas germline-specific genes, such as Dazl, Dppa3 and Vasa, are enriched for only H3K4me3 [20]. While in FGSCs, the developmental genes (e.g. OCT4) were occupied by H3K27me3 [21]. A tight link between OCT4 and H3K27me3 has been widely demonstrated [22,23]. For example, the pluripotency genes, including OCT4, were increased in EED knockout mESCs [24]. In the present experiment, there was elevated OCT4 expression upon artificial PRC2 suppression, indicating that the OCT4 expression was regulated by PRC2 in FGSCs. Those results further verify the above conclusion.

OCT4 is extensively expressed in ESCs and PGCs; however, it is expressed at low levels upon differentiation during mouse embryonic development [25]. In female PGCs, OCT4 is repressed by the onset of meiotic prophase I (E14.5) and re-expressed during the growth phase of oocytes after birth [26]. A previous study demonstrated that OCT4 deletion in PGCs resulted in apoptosis of early germ cells [27]. In addition, another study revealed that inhibition of Otx2 as a repressor for OCT4 could promote the expression of OCT4 and further increased the generation of PGCLCs derived from ESC [28]. Combined with these results, it is indicated that OCT4 is required for germ cell survival and proliferation. Similarly, OCT4 is also ubiquitously expressed in FGSCs. In multiple species, accumulating evidence supports the existence of FGSCs in neonatal and adult ovaries [29–32]. FGSCs, derived from either neonatal or adult mouse ovaries, could differentiate to form functional oocytes after transplantation into mouse ovaries or construction of ovarian organoid model [13,15,33]. While the two populations of FGSCs express OCT4, they differ from each other in expression pattern of OCT4. OCT4 appeared to be expressed in FGSCs derived from neonatal ovaries with a nuclear localization [29,33], and slightly in FGSCs derived from adult ovaries with a cytoplasm [34,35]. In this study, we used FGSCs derived from neonatal ovaries as the experimental subject to explore the potential effects of PRC2. The results indicated that inhibition of PRC2 promoted OCT4 expression and further demonstrated OCT4, as a crucial determinant that regulated the survival and proliferation of FGSCs. Notably, FGSCs with higher expression of OCT4 retained the normal germline capacity. We harboured the idea that this phenomenon might be attributed to several reasons. First of all, it has generally been accepted that pluripotency is regulated by a complex interconnected signalling network that is cooperatively regulated and maintained by several core pluripotency factors [36]. In the present study, our finding indicated that the expression of OCT4 was increased by inhibition of PRC2, and other pluripotency factors, including Nanog, Sox2 and Esrrb, were not statistically affected. Moreover, while OCT4, as a maternally inherited factor, has typically been detected in mature oocytes, its primary function is in the maintenance of germ cell proliferation and survival rather than classical germline determinants [37]. Thus, we consider that elevation of OCT4 alone is not sufficient to alter germline capacity.

The activation of P53 and P63 has an important impact on various developmental processes, such as DNA damage repair, cell differentiation, apoptosis and proliferation [38]. The abnormal elevation of activated P53 causes a complete loss of fetal germ cells during mouse embryogenesis [39,40]. Additionally, the absence of P63 could effectively block the apoptosis caused by ionizing radiation in PGC [41]. In this study, we found that the expression levels of P53 and P63 were significantly decreased by elevated OCT4 expression after inhibition of EED. By contrast, when OCT4 was knocked down by RNAi, P53 and P63 showed a significant elevation. This suggests that OCT4 plays a role in negatively regulating P53 and P63 expression. Studies indicated that OCT4 plays an important role in enhancing reprogramming efficiency and maintenance of the multi-/pluripotency of ESCs [42] and iPSCs [43] by suppressing the expression of P53. However, there is no consensus on the association between OCT4 and P63. Our results suggested that OCT4 has been shown to inhibit P53 and P63 expression in FGSCs, which in turn enhanced cell survival, while a comprehensive regulatory mechanism is still needed for further study.

In the germ cell development, FOA is a conserved phenomenon *in vivo* [44] and *in vitro* [33], occurring during the progression through the meiotic prophase I stages and the formation of primordial follicles. FOA, as a high-quality control system of oocyte selection, serves an important role during the establishment of the mammalian ovarian reserve [45]. The quality control system of the oocyte is essential for genetic inheritance stability. Any oocytes with DNA damage are removed by programmed cell death (PCD) [46,47]. During this process, a significant drop in the number of oocytes has been previously reported between E15.5 and E18.5, followed by a lesser loss of oocytes between E18.5 and within a few days of birth [48–50]. According to research, P63, as the principal member of the P53 family, is thought to play a role in a conserved mechanism for controlling female germline integrity [51,52]. In mice and humans, P63 expression begins in late pachytene-stage oocytes and peaks in diplotene oocytes. P63-null mice exhibited a high survival rate of oocytes [51] as well as the abnormal enrichment of γ-H_2_A (as an indicator of DNA damage) [53]. Another study demonstrated that double deletion of P53 and P63 can salvage oocytes that have been lost owing to checkpoint depletion, and the recovered oocytes are functional. After two months, the ovaries had a significant amount of all follicle types as well as recombination-defective oocytes [54]. In this experiment, we observed that the FGSCs with EED226 treatment yielded a larger number of follicles during oogenesis *in vitro* compared with control, accompanied by the low expression levels of P63 in the early stage of IVD culture. These results point to a synergistic role for P63 in controlling germ cell survival. Notably, similar to the *in vivo* results, concomitant with the reduction in P63 expression, ovarian organoids showed a higher proportion of recombination-defective oocytes, indicating the meiosis checkpoint role of P63 in oogenesis during reconstitution of mouse oogenesis from FGSCs.

Organoid is a new model that has a lot of potential for clinical applications. Hikabe *et al.* [55] and Yoshino *et al.* [56] established the reconstitution *in vitro* arising from ESCs or iPSCs, and the resultant oocytes produced healthy pups. Meanwhile, Luo *et al.* [15] and Li *et al.* [33] developed and described a female germline stem cell-derived ovarian organoid model. It also confirmed that the model endocrine function remained intact. Furthermore, primordial germ cell-like cells (hPGCLCs) were created by co-culturing human pluripotent stem cells (hPSCs) with mice ovarian somatic cells, and eventually developed into human oocyte-like cells [57]. Thus far, the three ovarian organoid models were basically established. In the present study, the research was based on the second model and performed optimally. It effectively improves the culture efficiency and yield of FGSCs via inhibition of EED activity. Further, we confirmed that inhibition of EED function did not affect the unipotency of FGSCs. This culture system used here will facilitate the study of potential mechanisms during mammalian oogenesis as well as provide clues to reproductive medicine.

Our result shows the successful establishment of an effective culture strategy to expand FGSCs and obtain oocytes *in vitro*, which is a reproducible tool that can be used for simulating the underlying mechanisms of oogenesis *in vitro*. Actually, ovary tissue possesses other stem cells yet, for example, VSELs, very small embryonic-like stem cells with nuclear OCT4 expression [34,58–62]. Multiple studies have revealed that VSELs had the potential for oocyte differentiation [60,61,63]. Our study may provide novel insights into VSEL expansion.

## 4. Material and methods

### 4.1. Animal breeding

The outbred ICR mouse was purchased from SPF Biotechnology (Beijing, China). β-Actin-GFP mice were donated by Lin Liu Lab (Nankai University, Tianjin, China). Mice were bred in the mouse house of Inner Mongolia University in a standard temperature/humidity constant environment.

### 4.2. Chemicals

EED226 (S8496) and GSK343 (S7164) were obtained from Selleck.com. As one of the inhibitors of PRC2, EED226 directly binds to the PRC2 binding pocket in EED226 [64]. Besides, GSK343 specifically inhibits the activity of EZH2 [65].

### 4.3. FGSC extraction and culture

In this study, the protocols for sorting FGSCs were identical to that used in previous studies [66,67]. In brief, ovaries from 1–3 dpp female mice were converted into single-cell suspension, then FGSCs were purified by the antibody against Fragilis (PA5-34598, Thermo, USA) and Dynabeads M-280 Sheep anti-Rabbit IgG (11203D, Thermo, USA). Sorted FGSCs were cultured in MEM-α (12561056, Thermo, USA), which was supplemented with 10% fetal bovine serum (10099, Gibco, USA), 2 mM Gluta^max^ (35050061, Gibco, USA), 30 mg ml^−1^ pyruvate (11360070, Gibco, USA), 1 mM nonessential amino acids (11140050, Gibco, USA), 5 mg ml^−1^ penicillin–streptomycin (10378016, Gibco, USA), 10 ng ml^−1^ recombinant human FGF-basic (100–18B, PeproTech, UK), 40 ng ml^−1^ human glial cell line-derived neurotrophic factor (RP-8602, Thermo, USA), 10 ng ml^−1^ mouse epidermal growth factor (E5160, Sigma, USA), 10 ng ml^−1^ mouse leukemia inhibitory factor (A35935, Gibco, USA) and β-mercaptoethanol (M6250, Sigma, USA).

### 4.4. CCK8 assay

FGSCs with 5 × 10^3^ cells per well were plated in 96-well plates. After cell culture for 48 h, CCK8 reagent (C0038, Beyotime, China) was added to the 96-well plate (10 µl well^−1^) and incubated accordingly. Finally, the absorption value was measured with a microplate reader (Bio-Tek Instruments, Thermo, USA) at 450 nm wavelength.

### 4.5. EdU staining array

FGSCs were seeded in 48-well plates, with 1 × 10^4^ cells per well. After 24 h, the cells were then incubated with fresh medium containing 10 µM EdU solution (C0071S, Beyotime, China) for another 2 h. FGSCs were fixed for 30 min in 4% paraformaldehyde and permeabilized with 0.5% Triton X-100 for 20 min. Then, according to the manufacturer's protocol, FGSCs were reacted with Click Additive Solution for 30 min, after which cells were treated with Hoechst solution for 10 min, and visualized under a fluorescent microscope. The percentage of EdU-positive cells was calculated by the following formula: EdU-positive rate = EdU-positive cell count/(EdU-positive cell count + EdU-negative cell count) × 100%.

### 4.6. RNA isolation and RT-PCR

FGSCs were pelleted by centrifugation to remove the extra medium and were then resuspended in 100 µl of RNAiso Plus (9109, Takara, Japan) for RNA extraction. A total of 1 µg of total RNA per sample was reverse transcribed into cDNA via a PrimeScript RT reagent kit with gDNA Eraser (RR047A, Takara, Japan). The primer's details are shown in electronic supplementary material, table S1.

### 4.7. Immunohistochemical staining

The fixed rOvaries were embedded in paraffin. 3–5 µm of paraffin sections were used for immunohistochemical assay. After the standard procedures of dewaxing, rehydrating and antigen repair, the slides were treated with 3% hydrogen peroxide in PBS to inactivate endogenous peroxidase activity and incubated with blocking buffer (10% serum in PBS), for 1 h at 37^°^C. After that, the slides were incubated with the first antibody overnight at 4^°^C, followed by the secondary antibody (HRP conjugated anti-rabbit IgG, A0279, Beyotime, China) incubation for 30 min at room temperature. HRP activity was detected with DAB solution (P0203, Beyotime, China). The slides were examined under a microscope and photos were taken for analysis by ImageJ. Antibodies and concentrations are listed in electronic supplementary material, table S2.

### 4.8. Three-dimensional culture

The recombinant ovary (rOvary, ovarian organoid) was produced according to a modified dynamic co-culture method [33,55,68]. A brief description is given below. FGSCs (from β-actin-GFP female mice ovary) were co-cultured with female gonadal somatic cells (from 1–3 dpp wild-type mice ovaries, from which germ cells have been removed by Ddx4 antibodies conjugated with magnetic beads) with GK15 + RA medium in a U-bottomed 96-well plate for 2 d. rOvaries were then transferred onto Transwell-COL membranes (3492, Corning, USA) soaked in GK15 + RA medium for 2 d. Afterward, rOvaries were cultured with IVD (*in vitro* differentiation) medium on 21 d and formed individual follicles. After this, the individual follicles were cultured with IVG (*in vitro* growth) medium for 11–14 d.

### 4.9. Western blot

FGSCs were collected and lysed in RIPA (P0013B, Beyotime, China) supplemented with a protease inhibitor cocktail to protein extraction. The concentration of the protein was measured by using the BCA protein assay (23225, Thermo, USA). 20 µg of proteins from each sample were mixed with 2 × loading buffer (P0015B, Beyotime, China) and then the assay performed following standard procedures. Bands were visualized with Clarity Western ECL Substrate (32209, Thermo, USA) and quantified with ImageJ. Antibodies and concentrations are listed in electronic supplementary material, table S3.

### 4.10. ChIP-qPCR assay

Briefly, FGSCs (10^4^ cells) with or without EED226 treatment were cross-linked, lysed, and sheared to obtain 200–800 bp fragments. Nearly 2 µg of either anti-H3K27me3 (ChIP-grade, 9733S, Cell Signaling Technology, USA) or anti-IgG (ChIP-grade, 2729S, Cell Signaling Technology, USA) was used for the immunoprecipitation reaction. Purified immunoprecipitated DNA with equal volumes were used in qPCR reactions (TB Green Premix Ex Taq, RR420B, Takara, Japan) with qPCR primers targeting the promoter, and exon regions of candidate genes. Electronic supplementary material, table S1, lists the ChIP-qPCR primers.

### 4.11. Lentiviral transduction

Lentiviral interference vectors, pLLU2G-*Oct3*-EGFP (*Oct3*, known as *Oct4*), were purchased from Addgene (21616, Addgene, China). For lentivirus infection, FGSCs were incubated with a mixture of culture medium and the lentivirus-concentrated solution containing 5 µg ml^−1^ polybrene. Empty vector (pLLU2G) transfected cells were used as controls.

### 4.12. Statistical analysis

All experiments were repeated at least three times. Experimental data were expressed as mean ± s.d. or SEM with each experiment, analysed by two-tailed Student's *t* test or one-way ANOVA, and considered statistically significant at values of *p* < 0.05.

## Data Availability

The datasets used and/or analysed during the current study are available from the corresponding author on reasonable request. Supplementary material is available online [69].
